# *Dientamoeba fragilis* cysts and pre-cysts in historic slide collections and a review of cyst formation among the parabasalia

**DOI:** 10.1017/S0031182026101942

**Published:** 2026-04

**Authors:** Luke M. Hall, Sarah Sapp, Mark Fox, Joel L. N. Barratt, John Timothy Ellis, Damien Stark

**Affiliations:** 1 School of Life Sciences, University of Technology Sydney, Broadway, NSW, Australia; 2 Division of Microbiology, Sydpath, St Vincent’s Hospital, Darlinghurst, NSW, Australia; 3 Division of Parasitic Diseases and Malaria, US Centers for Disease Control and Prevention, Atlanta, GA, USA

**Keywords:** cyst, *Dientamoeba fragilis*, historical slides, microscopy, transmission

## Abstract

*Dientamoeba fragilis* transmission is a basic aspect of this intestinal parasite’s biology that is poorly understood. Early historical reports reflecting the absence of a cyst are often cited as a central argument in debates supporting the lack of a *D. fragilis* cyst. While *D. fragilis* cysts have been described since Dobell’s original description, their existence is not universally accepted. Here, Dobell’s, Wenyon’s and Hoare’s collection of historical faecal smears stored at the Natural History Museum (London), dating back to the 1890s and the early 1900s, was examined for forms consistent with modern descriptions of *D. fragilis* cysts, and an example was found in 1 slide. Such rare forms were also detected during examination of stained faecal smears archived in the parasite reference laboratory collection at the United States Centers for Disease Control and Prevention. We discuss these observations in the context of literature describing cyst formation in parabasalids. Additionally, we report some incidental findings from past immunofluorescence experiments on cultured *D. fragilis*, which suggest differential staining that appears to correlate with life cycle stages. Considering published literature on the subject of *D. fragilis* cysts and the broader picture of cyst formation across diverse members of Parabasalia, we recommended that future investigations on *D. fragilis* transmission consider mounting evidence for the role of a true cyst despite its rarity in human faecal specimens. The factors leading to cyst formation and further characteristics of this life cycle stage require further study.

## Graphical abstract

**Figure:**
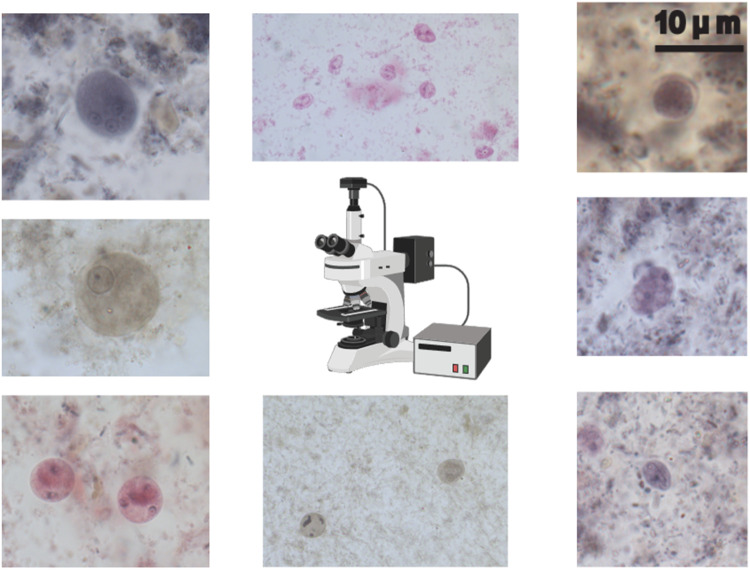

## Introduction

Investigators on the life cycle of *Dientamoeba fragilis* have proposed 3 possible modes of transmission for this intestinal protozoan: direct transmission via the trophozoite stage, transmission via a cyst stage (Munasinghe et al. 2013; Stark et al. 2014; Garcia, 2016; Hall et al. 2024) and transmission via an *Enterobius vermicularis* ova ‘vector’ that affords fragile trophozoites resistance to environmental stressors (Burrows and Swerdlow, 1956; Ogren et al. 2013; Roser et al. 2013). Experiments on cultured *D. fragilis* do not support a direct route of transmission for the trophozoite, as it cannot survive the acidic conditions of the human stomach (Hall et al. 2024). On this basis, the 2 alternative transmission models – via a cyst stage or via *E. vermicularis* ova (Clark et al. 2014; Hall et al. 2024) – seem more likely. However, neither model is universally accepted. Discussions supporting the lack of a *D. fragilis* cyst often cite Dobell’s rigorous microscopic observations that support its absence from the life cycle (Dobell, 1940; Clark et al. 2014). Regardless, while cyst stages are generally uncommon among the Parabasalia, true cyst stages have been noted in multiple species (Farmer, 1993; Hampl et al. 2007).

Perhaps the main challenge to the wider acceptance of a cyst stage in the *D. fragilis* life cycle is its rarity in human stool (Stark et al. 2014). However, evidence for the existence of *D. fragilis* cysts is mounting with increasing numbers of detailed descriptions available for reference (Munasinghe et al. 2013; Stark et al. 2014; Garcia, 2016; Hall et al. 2024). These studies describe true *D. fragilis* cysts as possessing the same fragmented karyosome as the trophozoite, a key feature that distinguishes *D. fragilis* from other protozoa. However, cysts are markedly smaller than trophozoites (∼5 µm versus ∼15 µm in diameter, respectively) and possess a thickened, electron-dense cyst wall (Munasinghe et al. 2013; Stark et al. 2014; Hall et al. 2024). A peritrophic space separates the cyst wall from the encysted parasite as described from transmission electron micrographs and light microscopic images taken from stained faecal smears (Munasinghe et al. 2013; Stark et al. 2014). *Dientamoeba fragilis* pre-cysts represent an intermediate between the cyst and trophozite. For example, pre-cysts possess the beginnings of a cyst wall and are smaller than trophozoites with a cytoplasm that is more densely staining in stained faecal preparations, although not as densely staining as mature cysts (Munasinghe et al. 2013; Stark et al. 2014). As the morphological features of this structure are clearly defined, it stands to reason that the elusive *D. fragilis* cyst might now be more readily recognized in human faecal smears. Moreover, as most reports on these structures have surfaced in recent decades, it seems likely that *D. fragilis* cysts are present in historic material (i.e. faecal smears prepared by earlier investigators) but were unintentionally overlooked due to their infrequent occurrence, lack of a clear and consistent morphological description and the absence of experimental evidence. The present work seeks to build on this body of evidence.

Here, historical faecal smears containing *D. fragilis* were examined, including those prepared by distinguished researchers Clifford Dobell, Charles Wenyon and Cecil Hoare, for forms consistent with *D. fragilis* cysts. We also examined decades-old faecal smears from slide collections within the parasite reference laboratory at the United States Centers for Disease Control and Prevention (CDC), including slides generated circa 1980s through the early 2000s from the donated collection of Professor Emeritus Lawrence R. Ash (University of California, Los Angeles), and present some incidental immunofluorescent observations supporting that cyst formation might occur in *D. fragilis* cultures, albeit very rarely. Finally, we compare *D. fragilis* cysts from these historic slide collections to *D. fragilis* cysts observed in experimentally infected rodents and to examples of binucleate cysts from other trichomonads.

## Methods

### Examination of historic stained faecal smears

Permanently stained smears (e.g. iron-haematoxylin and trichrome) known to contain *D. fragilis* were examined for the presence of trophozoites, pre-cysts and cysts. Slides retained at the Natural History Museum, London, were examined under brightfield microscopy using a Leica DM 5000 B microscope. Slides within the CDC’s collection were examined on an Olympus BX41 microscope, and images were taken using Olympus CellSens (version 2.2) software. Slides were first scanned using the 40× objective, and the 100× objective was used to assess the morphology of the forms observed in greater detail and to capture high-resolution images.

### Immunofluorescent staining

Cultured *D. fragilis* was subjected to an immunofluorescent staining technique developed in our laboratory to stain the surface of *D. fragilis* trophozoites. Isolates of *D. fragilis* were cultured from stool samples as part of a previous study in a medium modified from Barratt et al. (2010), composed of an inspissated serum slope overlaid with phosphate-buffered saline and supplemented with 3–5 mg of rice starch. Indirect immunofluorescent staining was performed as described in Supplementary File S1. Briefly, the method utilized a rabbit polyclonal antibody to bind the surface of *D. fragilis*, and a secondary anti-rabbit IgG antibody (raised in goats) conjugated to fluorescein isothiocyanate (FITC) (Sigma Aldrich, product number F0382). Slides containing immuno-stained parasites were cover-slipped and examined for fluorescence on an Olympus BX51 fluorescent microscope at 1000× magnification (i.e. under oil emersion) by exposing them to the excitation spectra for FITC (455–500 nm). Each immunofluorescent staining experiment was accompanied by negative control slides, including fixed unstained parasites (i.e. to exclude autofluorescence), and slides were treated only with the secondary antibody.

## Results

### Faecal smears examined at the Natural History Museum, London

**Figure 1.**
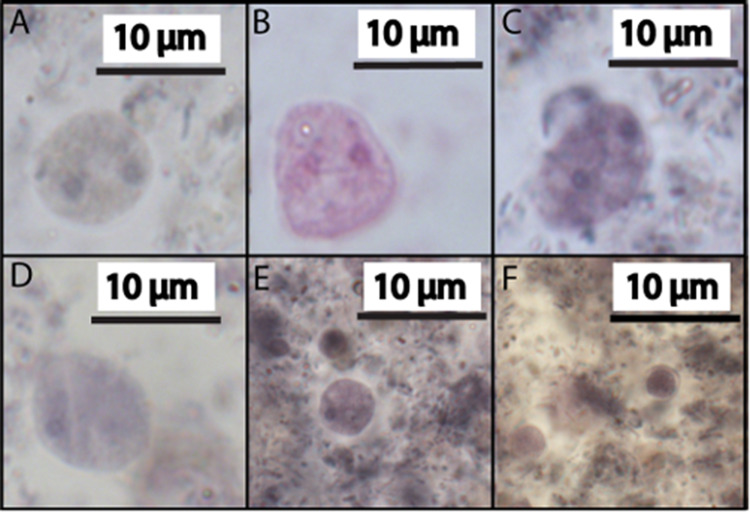
Micrographs of *D. fragilis* taken at the Natural History Museum, London. Figure 1 long description.

Protozoa consistent with *D. fragilis* were observed in the 17 preserved faecal smears examined at the Natural History Museum, including pleomorphic, typically binucleate trophozoites of 10–15 µm in diameter, and possessing a fragmented karyosome: a characteristic feature of *D. fragilis* (Figure 1 A–D). Catalogue numbers for each slide are provided in Table S2 of Supplementary file S1. Forms consistent with *D. fragilis* pre-cysts (Stark et al. 2014) were approximately 5 µm in diameter (Figure 1E). In addition to *D. fragilis, Endolimax nana, Entamoeba coli, Blastocystis hominis* and *Chilomastix mesnili* were observed in this collection. A single form (∼4 µm in diameter) consistent with descriptions of *D. fragilis* binucleated cysts was observed in slide 1987.4.4.15263, which was originally prepared in 1934 (Figure 1F). The cyst had a distinctive, thickened cyst wall with a slightly irregular inner membrane encasing the encysted parasite, which was surrounded by an unstained peritrophic space. The morphology of the nucleus was unclear due to the cyst being embedded within a thick, darkly stained part of the smear. *Dientamoeba fragilis* trophozoites and pre-cysts possessing the correct nuclear morphology were observed in this same smear (Figure 1C–E). This single form was considered a presumptive *D. fragilis* cyst due to the inability to observe its nuclear morphology. In addition to *D. fragilis, E. nana* and *C. mesnili* were present in the smear.

Micrographs of *D. fragilis* trophozoites (A–D), precyst (E) and presumptive cyst (F) as viewed under oil emersion microscopy (1000× magnification). The presumptive *D. fragilis* cyst stage from a historic slide (1987.4.4.15263) (F). This presumptive cyst possesses a distinctive outer cysts wall and encysted pleomorphic trophozoite, with an unstained peritrophic space.

### Faecal smears examined at the CDC

**Figure 2.**
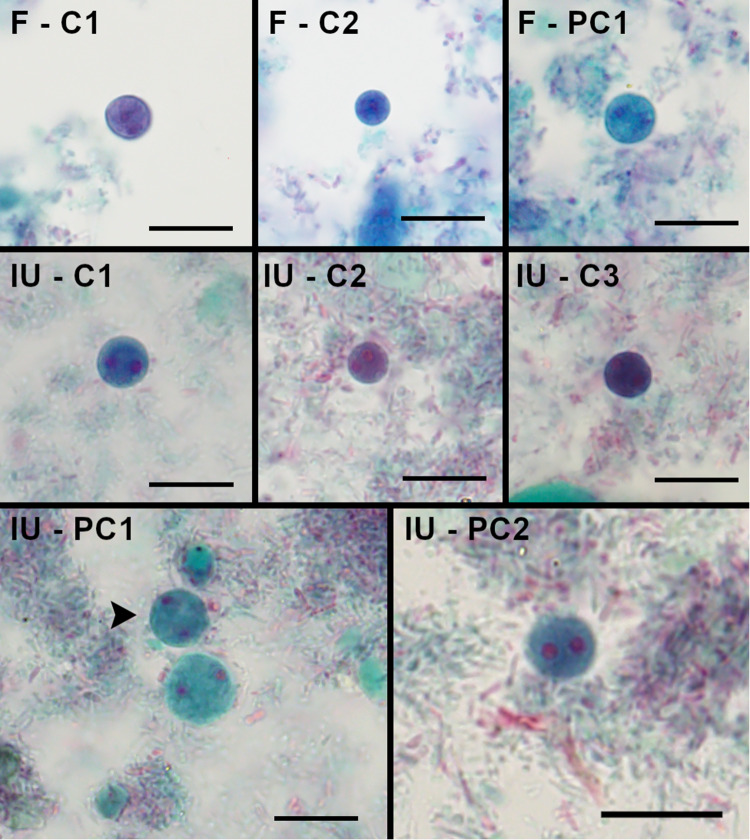
Micrographs of *D. fragilis* from trichrome-stained slides at CDC. Figure 2 long description.

**Figure 3.**
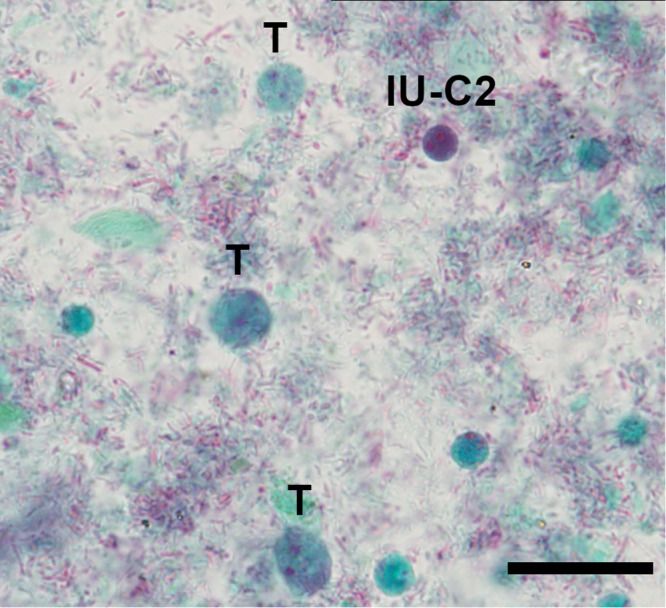
*Dientamoeba fragilis* cyst compared to *D. fragilis* to trophozoites and *Blastocystis* present on the same slide. Figure 3 long description.

**Figure 4.**
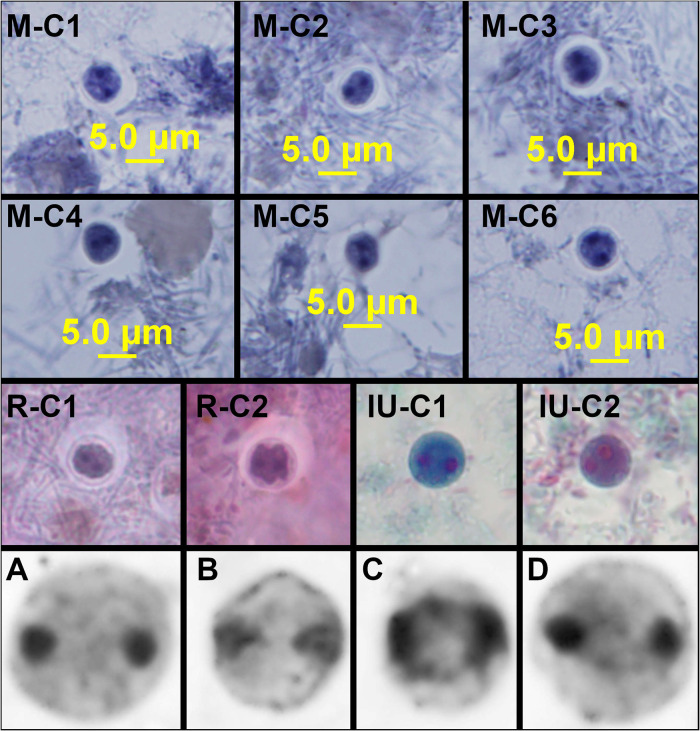
Examples of binucleate parabasalian cyst stages. Figure 4 long description.

**Figure 5.**
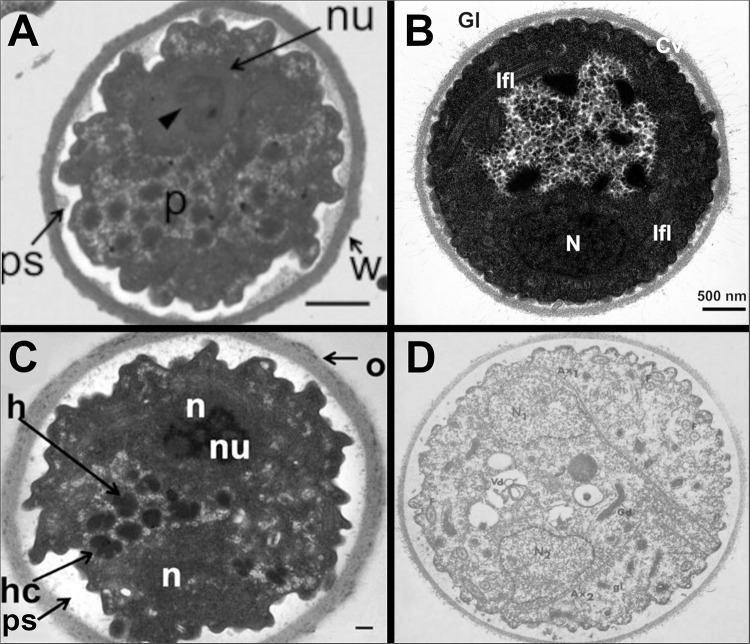
Transmission electron micrographs of parabasalian cyst stages. Figure 5 long description.

We detected forms compatible with the description of true cysts on 2 sets of permanently stained smears from 13 sets/series examined (Figures 2 and 3). All cyst forms were small (between 4 and 6.4 µm in diameter) and spherical with a thickened cyst wall and a lightly staining peritrophic space bounding the parasite within. Compared to trophozoites, the cytoplasm was darkly stained with a comparatively purple to blue hue and with a ‘smooth’, condensed appearance (Figures 2 and 3). Nuclear morphology was identical to the *D. fragilis* trophozoite stages. The appearance was consistent with that observed from infected rodents (Munasinghe et al. 2013) and to binucleate cyst forms described for other parabasalians (Figures 4 and 5). Transitional forms compatible with previously described pre-cyst stages were also present(Figure 2).

Top row: representative cysts (left, middle) and a pre-cyst (right) from slide F (see File S1 for a description). Middle and bottom row: representative cysts (middle row) and pre-cysts (bottom row) from slide set IU (see File S1 for a description). Panel IU-PC1 shows a pre-cyst (arrowhead), surrounded by a typical trophozoite below and *Blastocystis* above. Scale bars represents 10 µm. Slides viewed under oil emersion microscopy (1000× magnification). Dimensions of the organisms are shown and additional information about these slide sets are provided in Supplementary file S1 (Appendix D). Note that the *Blastocystis* in panel IU-PC-1 can be distinguished from *D. fragil*is by its large central vacuole and peripheral nuclei.

A representative *Dientamoeba fragilis* cyst (IU-C2) is shown in the same field of view as *D. fragilis* trophozoites (T) and several *Blastocystis* trophozoites (unlabeled). Note the relative size, shape and strength and hue of staining in the cyst compared to trophozoites. Typical nuclear morphology is shown in both the cyst and trophozoite forms and is distinct from that of *Blastocystis.* Note that cyst IU-C2 is also morphologically distinct from the *Blastocystis* trophozoites within this field. Scale bar represents 20 µm.

Panels M-C1 through M-C6: iron haematoxylin-stained smears generated in the study by (Munasinghe et al. 2013) from laboratory mice infected via ingestion of cultured *D. fragilis*. Panels R-C1 and R-C2: iron haematoxylin-stained smears prepared in the study by Munasinghe et al. (2013) from rats infected with *D. fragilis*. Panels IU-C1 and IU-C2 show a close-up of 2 cyst forms from Figure 2 magnified here for direct comparison to other forms in this panel. Bottom row: protargol-stained cysts of *Monotrichomonas transatlantica* (A and B), *Honigbergiellopsis adhaerens* (C) and *Honigbergiellida* sp. (D). (A) through (D) were reproduced from the paper by Céza et al. (2022).

(A) Electron micrograph of *D. fragilis* cyst reproduced from Munasinghe et al. (2013), showing the encysted parasite (p) within the outer cyst wall (w). The peritrophic space (ps), the nucleus (nu) and nucleolus (arrowhead) are also shown. Scale bar represents 1 µm. (B) Cyst of *Honigbergiella ruminantium* showing the cyst wall (Cv), nucleus (N), internalized flagella (Ifl) and glycocalyx (Gl). This image was reproduced from Hampl et al. (2007). (C) Electron micrograph of *D. fragilis* cyst reproduced from Hall et al. (2024), showing the outer cyst wall (o), the peritrophic space (ps), hydrogenosomes (h), ‘cauliflower-like’ hydrogenosomes (hc), between the margins of the 2 nuclei (n) and showing the nucleolus (nu) of one of the nuclei. (D) Cyst of *Trichomitus sanguisugae* showing classic parabasalian cyst features; spherical, binucleate, possessing a cyst wall and peritrophic space. This image was reproduced from Brugerolle (1973).

### Observations from immunofluorescence experiments

**Figure 6.**
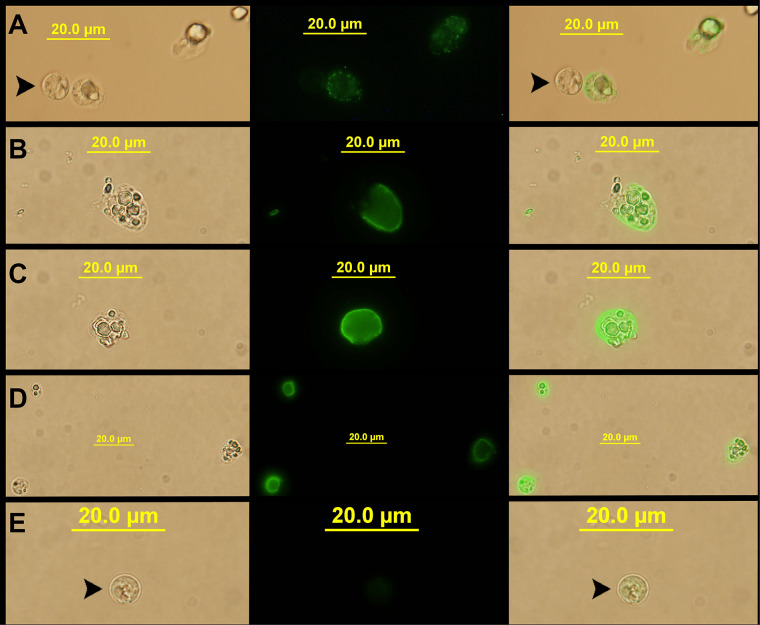
Indirect fluorescent antibody (IFA) staining of cultured *Dientamoeba fragilis.* Figure 6 long description.

While evaluating an immunofluorescent antibody (IFA) staining technique on cultured *D. fragilis* (Figure S3, File S1), we observed small (∼5 µm) spherical forms that failed to fluoresce following staining. At the same time, trophozoites within the same IFA preparations (sometimes within the same microscope field), stain readily (Figure 6). The proportion of rounded, unstained forms was very small versus typical trophozoites, but their presence was noted multiple times.

These micrographs show examples of cultured *D. fragilis* stained using an IFA method described here (Supplementary file S1). The first column shows *D. fragilis* under bright field microscopy, the second under fluorescent microscopy (FITC excitation spectra of 455–500 nm), and the third shows an overlay of the fluorescent signal in column 2 on the images in column 1. Row A shows forms on an IFA slide prepared with a sub-optimal primary antibody dilution of 1:10 (antibody B – see Supplementary File S1). Two of the 3 forms in row A show fluorescent staining and represent forms typical of cultured trophozoites (pleomorphic with ingested starch granules), while the third form (arrowhead) is spherical, approximately 5 µm in diameter, and failed to take on the fluorescent stain. Rows B through E are images of *D. fragilis* from 1 IFA slide prepared using an optimal primary antibody B concentration of 1:4 (as opposed to 1:10 for row A). Rows B through D show the characteristic staining of cultured trophozoites. Row E shows another spherical form of approximately 5 µm in diameter that does not fluoresce despite being observed on the same slide as the forms in rows B through D.

## Discussion

Here, we observed forms consistent with descriptions of true *D. fragilis* cysts in stained smears prepared by prominent investigators decades prior to the present work. These cysts of *D. fragilis*, although rare, are consistent and predictable in appearance and structure and are sufficiently distinct from trophozoites to warrant recognition as a genuine morphologic form. It also seems unlikely that *D. fragilis* cysts might be readily confused with *Blastocystis* or other protozoa by an experienced microscopist, even if both occur in the same specimen (Figure 3). The pre-cyst stage logically represents a transitional form between the trophozoite and cyst, marked by a rounded shape, a ∼50% reduction in size, and cytoplasmic changes indicated by darker staining (visible via iron-haematoxylin and trichrome) and a finer, less vacuolated cytoplasmic appearance. Additionally, our past experiments with cultured *D. fragilis* and passage of cysts into laboratory rodents (Munasinghe et al. 2013; Hall et al. 2024) also refute the idea that these morphologic changes correspond to terminal degeneration. Other workers who have successfully established cyst-forming trichomonads in culture have also observed transformation between the 2 forms (Farmer, 1993).

While characterization of *D. fragilis* cyst stages was not a focus of the immunofluorescent work described, the detection of small, spherical forms within *D. fragilis* cultures that failed to fluoresce seems noteworthy given the vibrant fluorescence displayed by trophozoites in the same preparations. The *D. fragilis* isolates used in these experiments were well characterized. They were first reported in 2010 (Barratt et al. 2010), were utilized in the rodent experiments from Munasinghe et al. (2013), were subjected to transcriptome sequencing (Barratt et al. 2015), and appeared in later work (Gough et al. 2020). These cultures had been examined microscopically by iodine wet preparation and via preparation of iron haematoxylin-stained smears for teaching and demonstration purposes on numerous occasions. While we cannot attest to the presence or absence of other eukaryotes in the original faecal material from which these cultures were derived (i.e. yeast and other protozoa), we can confirm that no other eukaryotes were present in these cultures at the time these works were performed. At that time, the cultures had been purified for *D. fragilis* by serial passage. This suggests that spherical structures that failed to fluoresce indeed belong to *D. fragilis* yet with a differential antigenic expression (perhaps associated with cyst wall formation) from trophozoites. A similar phenomenon has been reported for various other protozoal cysts (e.g. *Giardia, Entamoeba* and *Naegleria*), where differences in antigenic expression from the trophozoite result in differential antibody reactivity (Sparagano et al. 1993; Ratner et al. 2008; Kim et al. 2009; Spadafora et al. 2016). Therefore, we suggest that these spherical non-staining forms could represent *D. fragilis* cysts in culture; however, this is presumptive because no further efforts were made to investigate these forms beyond the work described. Further experiments are required to exclude alternative explanations for this observation, including antigen masking, fixation artefacts or cell cycle-related changes. Regardless, these observations seem worthy of reporting here in light of the discussion surrounding the *D. fragilis* cyst stage, lest they remain unpublished and become forgotten.

While *D. fragilis* cysts are rare in human feces, our observations support that these forms exist and may be observed in stained faecal smears on careful examination of such material. The immunofluorescence observations support that *D. fragilis* may also produce cysts in culture. These findings will challenge existing scepticism, although when viewed alongside earlier published observations (discussed below), the most parsimonious conclusion remains that these structures are true *D. fragilis* cysts. These entities are morphologically consistent rather than degenerate, binucleate with a fragmented karyosome – mirroring the trophozoite stage – and appear in faecal smears containing *D. fragilis* trophozoites. To argue otherwise would require postulating the existence of an as-yet-undescribed binucleate organism from human stool, possessing a nuclear structure identical to *D. fragilis*; a proposition many protozoologists might also find logically untenable.

Several arguments have been presented against the existence of the *D. fragilis* cyst: (1) that the rarity somehow undermines the status of it as a genuine morphologic/life cycle stage, (2) that these are simply misidentified or degenerate forms, (3) that trichomonads and other parabasalids do not form true cysts and (4) that the existence of cysts is incompatible with various proposed routes *D. fragilis* transmission. We believe these arguments are invalid based not only on our observations but also on the existing body of literature.

Firstly, it bears mentioning that the existence of true cysts among trichomonads is not a novel concept. The study of trichomonadid flagellates in general was notoriously challenging for historic protozoologists, marked by confusing nomenclature, varied quality of evidence and polarizing scholarly interpretations; the question of the formation of true cysts (i.e. a resting form with a discrete cell wall) in this group was long mired in this puzzle (Grassé, 1926). Prior to the 1970s, binucleate forms consistent with such cysts were described via light microscopy for a handful of parasitic trichomonads and related parabasalids, for example, *Trichomastix trichopterae* (Mackinnon, 1910), *Trichomitus batrachorum* (Dobell, 1908; Grassé, 1926) and *Trichomitus sanguisugae* (Alexeieff, 1911). Even still, many of these very workers remained circumspect and uncertain of the significance and origin of these forms due to their rarity, inconsistent recovery by other workers, case reports containing obvious misidentifications and insufficient descriptions. It was not until the advent of transmission electron microscopy that the genuine nature of these forms as true cysts – versus degenerate forms, artefacts or other protozoa – was confirmed, as shown by Brugerolle (1973) and later by additional authors.

The existence of *D. fragilis* cysts experienced a much similar progression and treatment. Since its original description by Jepps and Dobell in 1918, multiple workers have described putative cyst stages via light microscopy, but the general view was dubious of their significance, as reviewed in Stark et al. (2014) and Hall et al. (2024). For *D. fragilis*, Dobell’s conclusion against the existence of cysts was largely driven by this extreme scarcity in his study material, and this has remained central to modern debates on the existence of a true *D. fragilis* cyst. Some early reports of *D. fragilis* cysts (Wenrich, 1936) were explained away at the time as misinterpretations of degraded forms of the trophozoite stage (Dobell, 1940). Electron microscopy and other experiments were able to provide clarity as to the true nature of these cysts in the following decades.

This parallels the related concept and relevance of trichomonad pseudocysts, which have gradually gained acceptance as a legitimate life cycle phenomenon across several well-studied trichomonad parasites of warm-blooded animals, such as *Trichomonas vaginalis, Tritrichomonas foetus* and *Histomonas melagridis* (Pereira-Neves et al. 2003; Shiratori et al. 2023). While the pseudocyst phenomenon, involving rounded forms with withdrawn accessory structures, is distinct from the walled, true cysts and pre-cysts observed here, they both appear to serve the same function of survival in adverse conditions and in enhancing transmission. The ability to form a morphologically distinct resistant stage, whether as a pseudocyst or true cyst, appears to exist in a spectrum across parabasalids broadly. Perhaps this reflects evolutionary adaptations to a parasitic lifestyle, wherein the ability to form true cysts could have been lost for species that are highly adapted to transmission via close, direct contact between hosts. Farmer (1993) proposed that among the parabasalids, the transition from a free-living to parasitic lifestyle could have been mediated by accidental ingestion of walled, environmentally hardy cysts. The deep evolutionary relationships of the major parabasalid lineages remain poorly understood despite extensive analysis (Boscaro et al. 2024), and known cyst-formers do not form clean monophyletic clades. If ever achieved, further stable resolution of parabasalid phylogeny would provide valuable context to this hypothesis.

Observations and evidence from the other parabasalids that are known to form cysts support the legitimacy of the *D. fragilis* cyst. Céza et al. (2022) provide images of true cysts from *Monotrichomonas transatlantica, Honigbergiellopsis adhaerens* and *Honigbergiellida* sp. – reproduced here for direct comparison (Figure 4). True cysts have also been documented from *T. batrachorum* (Dobell and O’Connor, 1921; Brugerolle, 1973), *T. sanguisugae, Monocercomonas tipulae* (Brugerolle, 1973), *Ditrichomonas honigbergii* (Farmer, 1993) and *Honigbergiella ruminantium* (Hampl et al. 2007), each supported by electron microscopic descriptions (Figure 5). The monocercomonad cysts reported by Céza et al. (2022) are small (4–5 µm), spherical and binucleate, bearing likeness to binucleate cysts of *D. fragilis*. Similarly, electron micrographs of *D. fragilis* cysts from Munasinghe et al. (2013) and Hall et al. (2024) resemble those of *H. ruminantium* (Hampl et al. 2007) and *T. sanguisugae* (Brugerolle, 1973). As with *D. fragilis* cysts and the trichomonad cysts described by Céza et al. (2022), cysts of *T. sanguisugae* are binucleate (Brugerolle, 1973; Figure 5, panel D). Earlier work by Mackinnon (1910) describing *T. trichopterae* – a parabasalid from the alimentary canal of the caddis fly (*Trichoptera* sp.) – includes figures and descriptions of a cyst stage that is spherical, possessing a cyst wall, and with 2 nuclei that each display fragmented chromatin granules. On examining the phylogenetic position of various cyst-forming parabasalids, it is apparent that cyst production is not a monophyletic trait (Hampl et al. 2007; Boscaro et al. 2024).

The historic disagreement over *D. fragilis* cyst formation, coupled with the environmental fragility of the trophozoites, led to alternate hypotheses of transmission routes – namely, via infected helminth eggs, as is known for its close relative *H. melagridis*, which is capable of utilizing *Heterakis gallinarum* (caecal worm) as a ‘vector’ of sorts. For *D. fragilis*, the pinworm *E. vermicularis* has been proposed as fulfilling this role (Landim de Barros et al. 2022). This relationship is suggested by molecular evidence, where *D. fragilis* DNA was detected by PCR in DNA extracted from *E. vermicularis* ova that had been surface sterilized using hypochlorite solution (Ogren et al. 2013; Roser et al. 2013) and by some epidemiologic correlations (e.g. prevalence patterns in children and parents; Clark et al. 2014). The proposed pinworm egg-based transmission pathway is sometimes interpreted as precluding any possibility of cyst-based transmission (Clark et al. 2014). However, neither the laboratory nor the existing body of epidemiologic evidence is incompatible with the existence of *D. fragilis* cysts. It is also worth noting that *H. meleagridids* is capable of transmission by multiple routes (i.e. not exclusively via helminth ova), with its described pseudocyst or cyst-like form potentially playing a role in direct faecal-oral transmission (Munsch et al. 2009; Zaragatzki 2010a; Zaragatzki et al. 2010b; Beer et al. 2022).

Evidence supporting that *D. fragilis* produces a true cyst is growing, although it must also be acknowledged that these forms are rare and their relevance in transmission to humans is still largely unknown. Stark et al. (2014) estimated that cysts occur at a ratio of 1 for every 100 trophozoites, although the present work supports that they are generally rarer than this. Putative pre-cysts or forms described as ‘pseudocysts’ of *D. fragilis* were also reported by earlier investigators as being relatively uncommon (Kofoid, 1923; Kudo, 1926; Wenrich, 1936); one study reported such forms in 163 of 500 slides examined – 32.6% of patient samples (Stark et al. 2014). Early in the discovery of *D. fragilis,* Jepps and Dobell themselves proposed a potential explanation for their failure to detect cysts in the original description of *D. fragilis* – that the parasite may not be fully adapted to human hosts, and encystation may not occur normally in the human intestine. The influence of ‘subpar’ host species on the development of typical life cycle stages is not without precedence in the world of intestinal protozoa. For example, *Entamoeba histolytica* seems unable to form cysts in experimentally infected cats, which shed only trophozoites (Jepps and Dobell, 1918; Shimada et al. 1992; Roberts, 2025). In *D. fragilis*, cyst production does occur in humans, albeit rarely, which might suggest that the human gastrointestinal tract does not support the ideal conditions for efficient *D. fragilis* cyst development. In the experiments by Munasinghe et al. (2013), mice fed cultures of *D. fragilis* would readily excrete cysts, suggesting that the conditions of the murine alimentary tract are more conducive to cyst production than the conditions of the human alimentary tract. This could indicate that cysts are responsible for zoonotic transmission of *D. fragilis. Dientamoeba fragilis* has been detected in rats, budgerigars, pigs, cows, dogs, cats and non-human primates (Caccio et al. 2012; Chan et al. 2016; Jirku et al. 2022; Yetismis et al. 2022; Yildiz and Erdem Aynur, 2022), typically using molecular methods and sometimes without morphological support – though there are exceptions (Caccio et al. 2012; Yetismis et al. 2022). Given that the morphological features of the *D. fragilis* cyst are no longer obscure, molecular screening of faecal samples via PCR, followed by sequencing of amplicons, and subsequent microscopic examination of PCR-positive faeces might facilitate identification of additional hosts wherein *D. fragilis* cyst production occurs more readily.

Ultimately, the sporadic presence and rarity of *D. fragilis* cysts in human stool probably accounts for past difficulties in explaining the *D. fragilis* transmission cycle and justifies why even the preeminent historic parasitologists may have overlooked them (Dobell, 1940). It should be remembered that modern investigators benefit greatly from modern research tools. The technology required to produce and share the high-quality digital images shown here did not exist a century ago. The electron microscope was invented in 1931, and this technology was not widely used until decades later. Particularly relevant in this context was Brugerolle’s (1973) initial TEM-based characterization of the controversial cyst forms of various trichomonads, which had previously been limited to imprecise light microscopy-based descriptions and were thus considered dubious by many. With the advent of TEM technology, he was able to unambiguously confirm that these were not simply degenerating forms but possessed an ultrastructure compatible with true protozoal cysts. Subsequent similar work on related organisms, combined with modern collaboration and tools, has greatly benefitted this investigation–including descriptions and high-quality images obtained by Munasinghe et al. (2013) from *D. fragilis*-infected rodents and their TEM characterization (Munasinghe et al. 2013; Hall et al. 2024).

A limitation of the present study – and indeed most research on this topic – is the absence of molecular data to definitively link these structures to *D. fragilis*. The main challenge for the generation of such data is the rarity of these structures in stool, likely necessitating the use of laser capture microdissection and single-cell sequencing to ensure any recovered DNA is derived exclusively from these structures. Unfortunately, this would also destroy the original material. This was not an option for the present work given the historic nature of the slide collection. However, this means that the slides remain available to those who may wish to verify the present observations by examining the material for themselves. The immunofluorescence results might suggest that these forms could be purified from culture via flow cytometry for subsequent molecular analysis. Unfortunately, this was never pursued as it was not an objective of the immunofluorescent work, and the existence of these non-fluorescent forms was a purely incidental observation.

While *D. fragilis* cysts are rare in human faecal samples, the present study supports their existence and suggests they will be identified by microscopists who conduct a sufficiently diligent search. Consequently, prior assertions regarding the absence of a cyst stage in the *D. fragilis* life cycle should be reconsidered; future investigations into transmission must account for the mounting evidence supporting a true cyst stage. Importantly, the forms highlighted here are morphologically consistent rather than degenerate, binucleate with a fragmented karyosome and occur at low densities alongside *D. fragilis* trophozoites. Furthermore, these structures align with those previously observed in rodent models of *D. fragilis* infection and closely resemble the binucleate cysts of other trichomonads, as evidenced by various light and electron microscopic studies. As previously noted, to suggest these structures belong to an as-yet-undescribed protozoan rather than *D. fragilis* is a significantly less parsimonious explanation.

## Supporting information

10.1017/S0031182026101942.sm001 Hall et al. supplementary material Hall et al. supplementary material

## Supplementary material

The supplementary material for this article can be found at https://doi.org/10.1017/S0031182026101942.

## Figure 1 Long description

The image A showing one round, pale structure on a light background, with a black scale bar labeled 10 micrometer at the top. The image B showing one round structure stained light pink to purple with darker spots inside, on a light background, with a black scale bar labeled 10 micrometer at the top. The image C showing one round structure stained darker purple with clustered darker areas, on a light background, with a black scale bar labeled 10 micrometer at the top. The image D showing one faint round structure on a light background, with a black scale bar labeled 10 micrometer at the top. The image E showing multiple round structures of different sizes with darker staining and granular texture across the field, with a black scale bar labeled 10 micrometer at the top. The image F showing multiple round structures with uneven darker staining and granular texture across the field, with a black scale bar labeled 10 micrometer at the top.

Navigate back to Figure 1.

## Figure 2 Long description

The image A showing the label “F - C1” at the top left. A single round, dark purple structure is near the upper right. The background is pale with faint blue and pink staining. A black scale bar is at the lower right. The image B showing the label “F - C2” at the top left. A single round, dark blue structure is near the upper center. The background is pale with scattered light blue staining. A black scale bar is at the lower right. The image C showing the label “F - PC1” at the top left. A single round, blue structure is near the center. The background is pale with blue-green and light purple staining. A black scale bar is at the lower right. The image D showing the label “IU - C1” at the top left. A single round, purple structure is near the center. Small red dots are within the round structure. The background is pale with light blue and pink staining. A black scale bar is at the lower right. The image E showing the label “IU - C2” at the top left. A single round, purple structure is near the center. Small red dots are within the round structure. The background is pale with light blue and pink staining. A black scale bar is at the lower right. The image F showing the label “IU - C3” at the top left. A single round, dark purple structure is near the center. Small red dots are within the round structure. The background is pale with light blue and pink staining. A black scale bar is at the lower right. The image G showing the label “IU - PC1” at the top left. Three larger round, blue-green structures are grouped left of center. Small red dots are within the round structures. A black arrowhead is positioned to the left of the upper-left round structure. The background is pale with blue-green and light purple staining. A black scale bar is at the lower right. The image H showing the label “IU - PC2” at the top left. A single round, blue structure is near the center. Small red dots are within the round structure. A red elongated stained area is below and slightly left of the round structure. The background is pale with light purple and gray staining. A black scale bar is at the lower right.

Navigate back to Figure 2.

## Figure 3 Long description

The image features a person's hand holding a lit cigarette. The cigarette is the focal point, with smoke curling upwards. The background is blurred, suggesting a shallow depth of field. The hand appears to be holding the cigarette between the index and middle fingers.

Navigate back to Figure 3.

## Figure 4 Long description

The image A showing a circular grayscale field with two darker round areas positioned left and right of center. The image B showing a circular grayscale field with multiple darker areas, including a darker region near the upper-left edge and another near the right side. The image C showing a circular grayscale field with a darker round area near the left side and a broader darker region toward the lower-right. The image D showing a circular grayscale field with two darker round areas positioned left and right of center.

Navigate back to Figure 4.

## Figure 5 Long description

The image A showing a circular cell cross-section with a thick outer boundary and a granular interior. Text labels include “nu”, “p”, “ps” and “w”. Several arrows point inward from the perimeter toward the interior. The image B showing a circular cell cross-section with a dark outer rim and a dense, speckled interior containing multiple darker patches. Text labels include “GI”, “c”, “lfl” and “N”. A scale bar at the lower right reads “500 nm”. The image C showing a circular cell cross-section with a granular interior and multiple darker round areas. Text labels include “h”, “hc”, “ps”, “n” and “nu”. Several arrows point from the left side toward the interior. The image D showing a circular cell cross-section without label arrows. The interior contains many small, round and irregular light and dark regions. No text labels are present.

Navigate back to Figure 5.

## Figure 6 Long description

The image features a person's hand holding a lit cigarette. The cigarette is the main focus, with smoke curling upwards. The background is blurred, suggesting a shallow depth of field. The lighting highlights the burning tip of the cigarette and the hand holding it.

Navigate back to Figure 6.
