# Supplementary material for: Dientamoeba fragilis cysts and pre-cysts in historic slide collections and a review of cyst formation among the parabasalia
Source: Parasitology. 2026 Apr 13;153(5):716–25. doi: 10.1017/S0031182026101942 (PMC13315227; doi:10.1017/S0031182026101942)
Supplement: Hall et al. supplementary material [file S0031182026101942sup001.docx]

**Supplementary Methods:**

***Dientamoeba fragilis* cysts and precysts in historic slide collections and a review of cyst formation among the Parabasalia**

Luke M. Hall^1, 2*^, Sarah G. H. Sapp^3^, Mark Fox^3^, Joel L. N. Barratt^4^, John T. Ellis^1^, & Damien J. Stark^2^

**^1^** School of Life Sciences, University of Technology Sydney, Broadway, NSW 2007, Australia

**^2^** Division of Microbiology, Sydpath, St Vincent’s Hospital, Darlinghurst, NSW 2010, Australia

^3^Division of Parasitic Diseases and Malaria, US Centers for Disease Control and Prevention, Atlanta, Georgia, 30329, USA

^4^Department of Pathology and Laboratory Medicine, Emory University School of Medicine, Atlanta, Georgia, 30322, USA

*Correspondence:

[Luke.Hall@uts.edu.au](mailto:Luke.Hall@uts.edu.au)

E-mail Adresses

LH: [Luke.Hall@uts.edu.au](mailto:Luke.Hall@uts.edu.au)

SGHS: [xyz6@cdc.gov](mailto:xyz6@cdc.gov)

MF: [nyg3@cdc.gov](mailto:nyg3@cdc.gov)

JLNB: [joel.barratt@emory.edu](mailto:joel.barratt@emory.edu)

JE: [John.Ellis@uts.edu.au](mailto:John.Ellis@uts.edu.au)

DS: [damien.stark@svha.org.au](mailto:damien.stark@svha.org.au)

# Supplemental Methods

## Polyclonal antibody development

### Identification of *D. fragilis* α-enolase mRNA sequences

The α-enolase protein of *D. fragilis* was selected as a target for polyclonal antibody development given that α-enolase homologs from other microbes (including certain pathogenic protozoa) are surface associated and immunogenic ^1-5^. To identify α-enolase homologs from within the publicly available *D. fragilis* transcriptome ^6^, a fasta file containing all assembled *D. fragilis* transcripts was used to construct a standalone nucleotide BLAST database. An enolase homolog from *Trichomonas vaginalis* (GenBank accession: XM_001325471) was used as a query sequence in a BLASTN search against these *D. fragilis* transcript sequences. Putative *D. fragilis* enolase transcripts identified via this search were subjected to BLASTN and BLASTX searches against the NCBI non-redundant nucleotide and protein databases, respectively, to further confirm their identity. Putative *D. fragilis* enolase transcript sequences were translated *in silico* using the ‘Translate’ function of the ‘Sequence manipulation suite’ ^7^ (see: [link](http://www.bioinformatics.org/sms2/translate.html)), InterProScan (see: [link](http://www.ebi.ac.uk/Tools/pfa/iprscan/))) and NCBI’s 'Conserved Domains' feature (see: [link](https://www.ncbi.nlm.nih.gov/Structure/cdd/wrpsb.cgi)) were used to identify structural characteristics that would support the identity of these translated protein sequences as belonging to the enolase family.

### Peptide design and production of anti-peptide antisera

Peptides were designed using translated enolase protein sequences as a template. Briefly, the location of transmembrane helices and the topology of enolase sequences was predicted using the HMMTOP server (see: [link](https://hmmtop.pbrg.hu/html/submit.php)) ^8^. Peptides were designed from the predicted extracellular loop of enolase sequences using various tools. These included the Kolaskar & Tongaonkar Antigenicity prediction tool ^9^, the Bepipred linear epitope prediction tool ^10^, and tools available on the BCEPRED server ^11^. Consideration of peptides for synthesis was generally based on a consensus between the tools. If a cysteine residue was absent from either end of a selected native peptide sequence, a cysteine residue was added to one end to allow conjugation to keyhole limpet hemocyanin (KLH) via the cysteine thiol group. The thiol group is also essential for linking of the peptides to immuno-affinity purification columns (see below). Peptide synthesis and production of rabbit anti-peptide antisera was outsourced to the service provider MIMOTOPES (http://www.mimotopes.com/). The presence of anti-peptide antibodies in the rabbit antisera was confirmed via slot blot against the target peptides. Briefly, peptide was blotted onto a nitrocellulose membrane using a slot blot suction manifold, and the peptide was probed with the antisera (diluted 1:2 in PBS). Blots were then probed with an anti-rabbit IgG antibody (raised in goats) conjugated to FITC (Sigma Aldrich, product number F0382) (diluted 1:80). Blots were scanned using a Pharos FX Plus Molecular Imager (Bio-Rad) to determine the presence or absence of fluorescence. The blot was accompanied by two negative controls; a pre-bleed sample from the rabbit (prior to immunization with the peptide) and blots probed with the FITC conjugated antibody only (no rabbit anti-sera). Slot blot results were positive for each peptide supporting the presence of anti-peptide IgG antibody within the antisera.

### Affinity purification of antibody

Peptides were covalently linked to an agarose bead matrix (via their cysteine thiol group) for production of affinity columns using a SulfoLink® Immobilization Kit for Peptides (Thermo Scientific, product number 44999). Rabbit antisera were diluted 1:2 in PBS and the IgG fraction was precipitated using the saturated ammonium sulphate precipitation technique. The resulting suspension was centrifuged to pellet the protein (7000 g at 4°C for 15 minutes) and the pellet was dissolved in PBS. This solution was dialysed overnight at 4°C against 1L of PBS with three buffer changes. The solution was adjusted to contain an additional 300mM of NaCl. Affinity columns were equilibrated with a binding buffer (PBS plus an additional 300mM of NaCl). The antibody solution was sterile filtered (0.2μm filter) and applied to the column. Elution was performed using 0.2M glycine (pH 2.5). Eluted fractions were collected into tubes containing 400 μL of neutralisation buffer (1M Tris pH9). The absorbance of each fraction (at 280 nm) was measured to identify the IgG containing fractions. The IgG containing fractions were pooled and dialysed overnight at 4ºC against 1L of PBS with three buffer changes. The purity of antibody preparations was evaluated by reducing a portion with dithiothreitol (DTT) and subjecting it to polyacrylamide gel electrophoresis. Gels were stained with Coomassie blue for observation of IgG heavy and light chains.

## Dientamoeba culture

Trophozoites of *D. fragilis* were cultured at 37℃ under microaerophilic conditions from unpreserved human stool in a medium modified from Barratt, Banik ^12^. Briefly, this medium consisted of an inspissated horse serum slope overlaid with 5 mL of phosphate buffered saline (PBS) supplemented with 2-5 mg of rice starch. Cultures were passaged every 3 days as described elsewhere ^12^.

## Indirect fluorescent antibody staining of D. fragilis

Prior to immunofluorescent staining, *D. fragilis* trophozoites were either subjected to mild (non-permeabilizing) fixation or by fixation in sodium acetate acetic acid formalin (SAF). For the mild fixation, cultured *D. fragilis* trophozoites were bathed in PBS containing 3.7% formaldehyde and incubated for 10 minutes. Trophozoite cultures were also mixed with SAF (Fronine Lab Supplies, Australia) in a 1:1 ration and allowed to sit for at least 24 hours. After fixation, the tube was shaken gently to resuspend the cells. The trophozoite suspension was centrifuged at 700 g for 5 minutes and the supernatant discarded. Trophozoites were immediately bathed in a large volume of PBS and centrifuged once more to remove any residual fixative solution. Mayers albumin (40μl) was smeared on glass microscope slides. Fixed cells were then smeared onto the slide with the albumin and allowed to dry. Slides were blocked for one hour with 5% skim milk or 3% BSA followed by three washes in a solution of 0.2% BSA in PBS. Slides were then incubated in a solution of polyclonal antibody for one hour followed by three washes. Various concentrations of each polyclonal antibody (1:2, 1:4, 1:6, 1:8, and 1:10 diluted in PBS) were evaluated. Finally, the slides were incubated in a solution of dilute (1:80) FITC conjugated anti-rabbit IgG (Sigma Aldrich, product number F0382) for one hour followed by an additional three washes. The slides were cover slipped and trophozoites were examined for fluorescence under oil emersion microscopy using an Olympus BX51 microscope at 1000X magnification.

## Indirect fluorescent staining of other protozoa

### Cultivation of *Blastocystis* sp., *Giardia lamblia* and *Entamoeba* spp.

Clinical isolates of *Blastocystis* sp*.* were cultured from human stool specimens obtained at St Vincent’s Hospital, Sydney into a modified BD medium in accordance with the protocol described by Roberts, Barratt ^13^. A culture of *Entamoeba moshkovskii* (Tshalaia Strain – ATCC 50262) was cultured in the same biphasic media used for *D. fragilis* in this study, though with an added support flora consisting of *Escherichia coli* reference strain (ATCC 25922) used routinely at St Vincent’s hospital. Similarly, a strain of *Entamoeba histolytica* cultured from a clinical sample was grown the same media used for *D. fragilis* and *E. moshkovskii*. *Giardia lamblia* trophozoites were cultured axenically at 37℃ in TYI-S33 medium. *Blastocystis*, *Entamoeba* and *Giardia lamblia* were grown to log phase and subjected to the fixation and the fluorescent staining protocols described above for *D. fragilis*. Each *Blastocystis, Giardia* and *Entamoeba* IFAT was accompanied by a *Dientamoeba* positive control slide and negative control slides comprising *Blastocystis*, *Giardia* and *Entamoeba* stained with the fluorescent FITC conjugated antibody only.

# Supplementary Results

## Identification of D. fragilis enolase transcripts

Four *D. fragilis* contigs (CONTIG_9, CONTIG_13, CONTIG_582 and CONTIG_4111 –Appendix A) were identified as homologs of *T. vaginalis* enolase 4 (XM_001325471). The NCBI 'Conserved Domains' output indicated that translations of these contigs possessed an enolase C-terminal triosephosphate isomerase (TIM) barrel domain and a conserved enolase N-terminal domain. InterProScan also confirmed enolase N-terminal and C-terminal domains, and an enolase conserved site. Alignment of these sequences using Clustal W revealed that CONTIG_4111 and CONTIG_9 were almost identical (differing by several bases) although CONTIG_9 was marginally truncated by comparison. When CONTIG_9 and CONTIG_4111 were translated, their amino acid sequences were identical (referred to here as *D. fragilis* enolase ENO1). The translated protein sequences generated for CONTIG_13 and CONTIG_582 are referred to as ENO2 and ENO3, respectively (appendix B).

## Peptide design and polyclonal antibody preparation

Seven peptides were designed based on the three translated *D. fragilis* enolase sequences (Table S1). These were synthesized by MIMOTOPES and used for raising antisera (Figure S1). The quality of the affinity purified polyclonal antibody preparations raised using these peptides was confirmed by polyacrylamide gel electrophoresis under reducing conditions (i.e., following DTT treatment) to view the IgG heavy and light chains (Figure S1).

# Table S1. List of peptides designed in this study and their location within *Dientamoeba fragilis* enolase polypeptide sequences

| **Peptide name** | **Other name** | **Start functional group** | **Amino acid**  **sequence** | **End functional group** | **Location relative to full length protein sequences** |
| --- | --- | --- | --- | --- | --- |
| A | MLEE | Acetyl (Ac) | CLQIYEYLKEHDMLEE | Hydroxy (OH) | ENO1  ----------------------------------IGETTDPVGTLSRLLKAKAPAPTIDHLV  ENO2  -SLFLMADNKAASAEYLQEHKITELLQEAMNEMLATKPEDAFGFLARFFKTKAAAPVIDHLV  ENO3  VMSTQELTPAQKTQEYLEQYKIREVLQEAVNILLENKPEDAAGVLSTLMKNRALPPVVDHLV  ENO1  GREVLDSRGNPTVETDVYVNYLGTVMFAGRSSAPSGASTGSNEARELRDGGK-RYGGKGTQK  ENO2  GREVLDSRGNPTVETDVYVKYLGQVQFAGRSSAPSGASTGSNEARELRDSDSPRYQGKGTQK  ENO3  GRQVLDSRGNPTVEVDVYAKHLGEVVMAGCSSAPSGASTGSNEAKELRDGDPKKYCGKGTVT  ENO1  AAANVTNILSPALKGMKLDDLKAIDAKICATDG---TELKERVGGNACTATSFAIAEAAAAL  ENO2  AAHNVAEILSKALEGKKLDNLKELDEKICKADG---TELKENVGGNACTATSFALAETGAEL  ENO3  AAENVTKIISPALKQMSFESLQDLDRAICRLASNKDSELKEKVGGNATTASSFALATAAANL                        Peptide C                          Peptide D  ENO1  EEIPLFLYFARQYFGEQNMPKKYKLPQCCFNILNGGKHAGGNLKIQEFMASPAKGTPLPEQL  ENO2  EGLQLFEYLARQYYGAENVPKKFKLPTPFFNILNGGKHAGGNLKLQEFMASPAPGIAFPDQL  ENO3  NNEELFLYLARQYHN--EVPEKFKLPTPFCNILNGGKHAGGKLKIQEFMIAPREDLPFPEQL                                                Peptide B  ENO1  RMVAEVYQKLGAILVKEKGVSAKNLGDEGGFAPCLDTPDEAISYIEQAMKAAGYEPGKDIGL  ENO2  RMVAEVYQKLGGLLVKKYGLSAKNLGDEGGFAPLLNTPEEAISIIEEAIKEAGYEPGKDIRI  ENO3  RMVAEIYHVLGNILAKEKGVSAKNLGDEGGYAPLLDTPEEALTYIERAIVEAGYKPVEDVKL                Peptide F                Peptide E  ENO1  CLDAAASEFYDAETKLYEVEIGVKKTGDEMIQYWADLIAKHPCIISIEDGLDEKDYEHWAKL  ENO2  GLDAAASEFYDAETKLYEVEVDVKKTGDEMIQYWADLIAKHPCIVSIEDGLDEKDYEHWAKL  ENO3  GIDAAASEFYDETTKLYEIEVGVKKTGDEMIQYYKDLIAKHPAIVSIEDGLDEKDYKTWIKM    ENO1  NAALGDKVQLVGDDLYTTNPKTIAKGIEGKWCNALLLKVNQIGTISEAMQAAKLVLDAHQN  ENO2  NETIGDKVQLVGDDLYTTNPKTIAKGIEGKWCNALLLKVNQIGTISEAMQAAKLILDAHQN  ENO3  NKEIGDKIQLVGDDLYTTNPKTIQRGLKEKWCNALLLKVNQIGTITEAMRAARLVLEAGQK        Peptide G                                   Peptide A  ENO1  VMVSHRSGETCNSVIADLVVGIGAQQIKTGSTARGERIQKYTRLLQIYEYLKEHDMLEE  ENO2  VMVSHRSGETCNSVIADLAVAIGAQQIKTGSTARGERIQKYTRLLQIYEYLKEHDMLEE  ENO3  VMVSHRSGETCNSLIADLSVAIGAQYIKTGSLARGERIQKYTRLLQIYEYLKENEMLSE |
| B | SY | Acetyl (Ac) | CAISYIEQAM | Amine (NH_2_) |  |
| C | PK | Acetyl (Ac) | EQNMPKKYKLPQC | Amine (NH_2_) |  |
| D | SPAK | Acetyl (Ac) | CSPAKGTPLPEQ | Amine (NH_2_) |  |
| E | WADL | Acetyl (Ac) | WADLIAKHPC | Amine (NH_2_) |  |
| F | FYDA | Acetyl (Ac) | CFYDAETKLY | Amine (NH_2_) |  |
| G | GET | Acetyl (Ac) | VMVSHRSGETC | Amine (NH_2_) |  |

Notes: The peptide names listed here also correspond to the names given to the antibody preparations. For example, peptide A gave rise to antibody preparation A, peptide B gave rise to antibody preparation B and so forth. The location of each peptide relative to *D. fragilis* ENO1 is shaded black. Amino acids within each peptide that are conserved for all *D. fragilis* enolase paralogs are shaded grey.


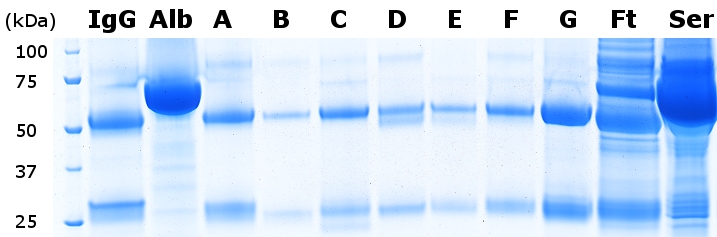


### Figure S1. Polyacrylamide gel electropherogram showing reduced antibody preparations A-G with controls.

(Lane ‘IgG’) Positive control. A commercially available polyclonal IgG antibody from rabbit (Abnova, Cat. No. PAB0918) reduced with DTT. (Lane ‘Alb’) Bovine serum albumin reduced with DTT. (Lanes A to G) Polyclonal antibody preparations A to G produced in this study, reduced with DTT. (Lane ‘Ft’) Example of ‘flow-through’ material after an affinity purification run, comprising proteins that failed to bind to the column. (Lane ‘Ser’) Whole rabbit serum reduced with DTT. Polyclonal antibody preparations A through G are pure and contain only trace amounts of albumin and other serum proteins. This figure also demonstrates that polyclonal antibodies A to G are of a similar high quality and purity to the commercially available antibody PAB0918 from Abnova.

## Indirect fluorescent antibody (IFA) staining of non-permeabilised trophozoites

The seven antibody preparations were evaluated for their ability to stain the surface of *D. fragilis* trophozoites. Antibodies raised against peptides A (optimum dilution 1:10) and B (optimum dilution 1:4) exhibited strong reactivity to the surface of *D. fragilis* trophozoites (Figure S1) following fixation in 3.7% formaldehyde in PBS and in SAF (Figure S2). Other antibody preparations produced a weak or absent fluorescent signal. Antibody preparations A and B gave rise to slightly different fluorescent patterns. Antibody A produced a diffuse, even fluorescent signal while antibody B produced a distinct ring pattern of fluorescence characterized by a densely stained fluorescent ring on the periphery of cells (Figure S2, Figure S3). Cells treated with only the secondary antibody did not fluoresce. Examination of fixed, unstained cells using an Olympus BX51 fluorescent microscope using the same FITC excitation spectra (455 - 500 nm) confirmed a lack of auto-fluorescent. Cells from control slides stained with antibodies A and B without the the FITC conjugated anti-rabbit IgG also failed to fluoresce.

# Indirect fluorescent staining of other protozoa

*Indirect fluorescent antibody staining of cultured parasites*

When *Giardia lamblia*, *Blastocystis*, *E. histolytica* and *E. moshkovskii* were stained using antibody preparation A, strong fluorescence was observed on the surface of *E. moshkovskii* and *E. histolytica* though *Blastocystis* cells failed to fluoresce (Figure S4). For Giardia, a speckling pattern of fluorescence was observed, suggesting reactivity to certain cellular components of *Giardia* trophozoites. When polyclonal antibody B used to stain trophozoites of *Entamoeba*, *Giardia lamblia*, and *Blastocystis*, no fluorescence was observed. It was concluded that antibody B exhibited greater specificity for *D. fragilis*.


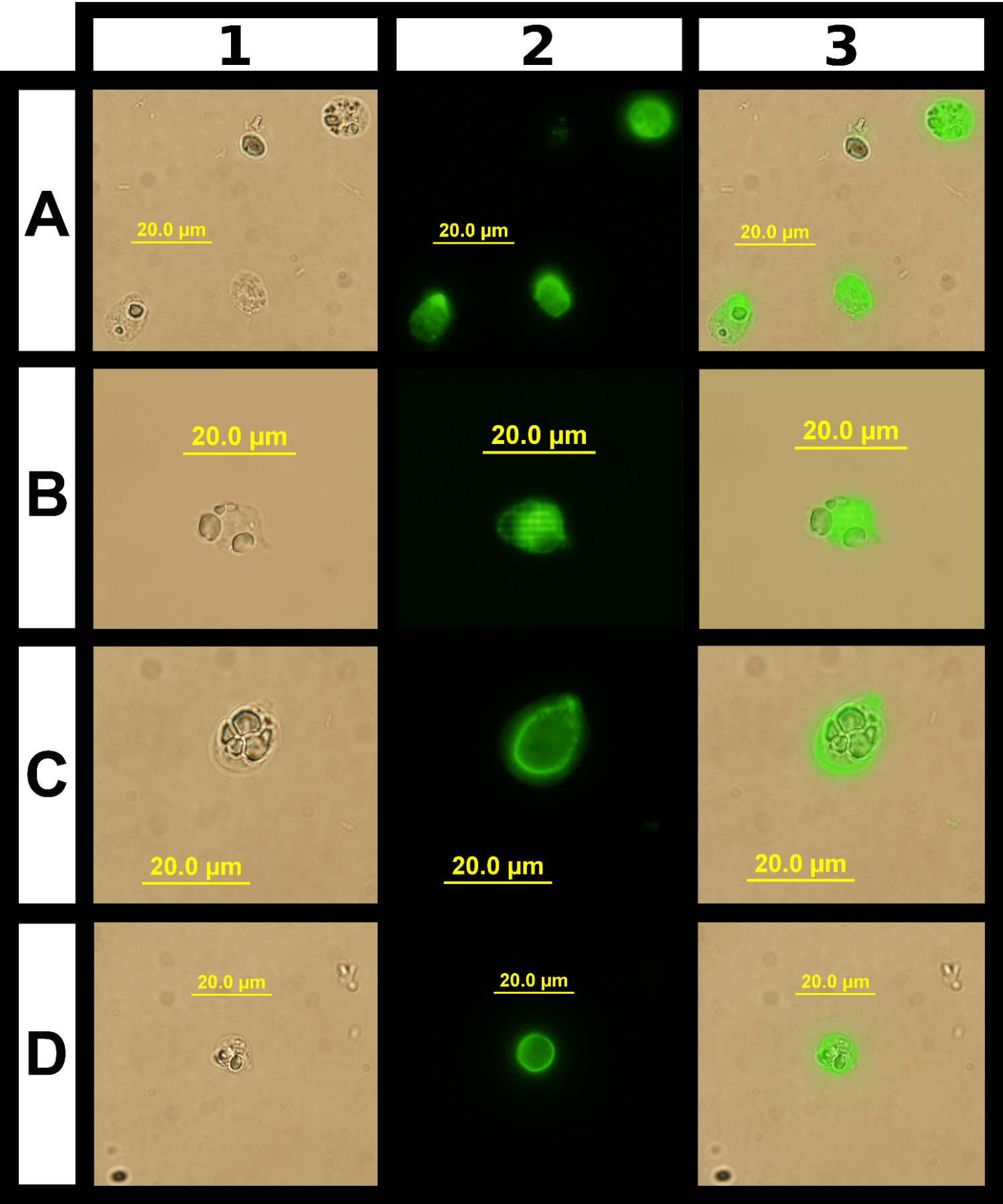


### Figure S2. Fluorescent micrographs of 3.7% formaldehyde fixed (non-permeabilised) *D. fragilis* trophozoites

Cultured *D. fragilis* trophozoites were stained using the indirect fluorescent antibody technique described here. Rows A and B show *D. fragilis* trophozoites stained using antibody A (optimal 1:10 dilution). Rows C and D show *D. fragilis* trophozoites stained with antibody B (optimal 1:4 dilution). Images in column 1 are bright field micrographs. Images in column 2 are the corresponding fluorescent micrographs. Column 3 represents an overlay of images from column 2 on column 1.


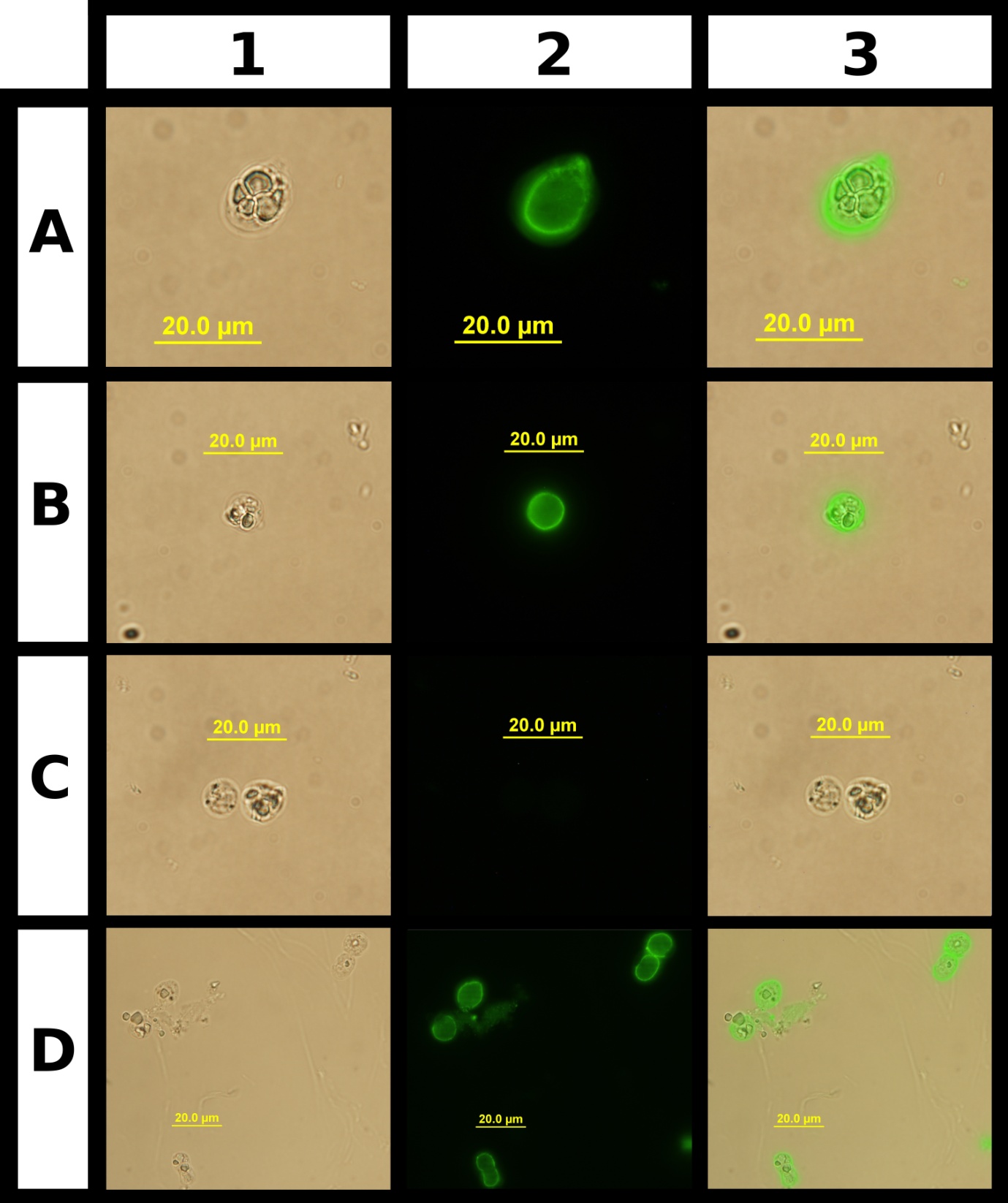


### Figure S3. Staining of *D. fragilis* trophozoites using polyclonal antibody B.

(Rows A and B) *Dientamoeba fragilis* trophozoites (the same shown in Figure S1 panels C and D), subjected to fixation in 3.7% formaldehyde and stained using polyclonal antibody B at a dilution of 1:4. (Row C) *D. fragilis* trophozoites from the corresponding negative control slide for rows A and B (trophozoites stained with only the secondary antibody [1:80 dilution]). The negative control image was over-exposed to ensure that fluorescence was completely absent. (Row D) Additional examples of *D. fragilis* trophozoites stained using antibody B fixed in SAF. Trophozoites in rows A, B and D display the distinct ring pattern which is typically observed when staining is performed using antibody B. (Column 1) Bright field micrographs. (Column 2) Corresponding fluorescent micrographs of the same trophozoites shown in column 1. (Column 3) Overlay of fluorescent micrographs on the bright field micrographs.


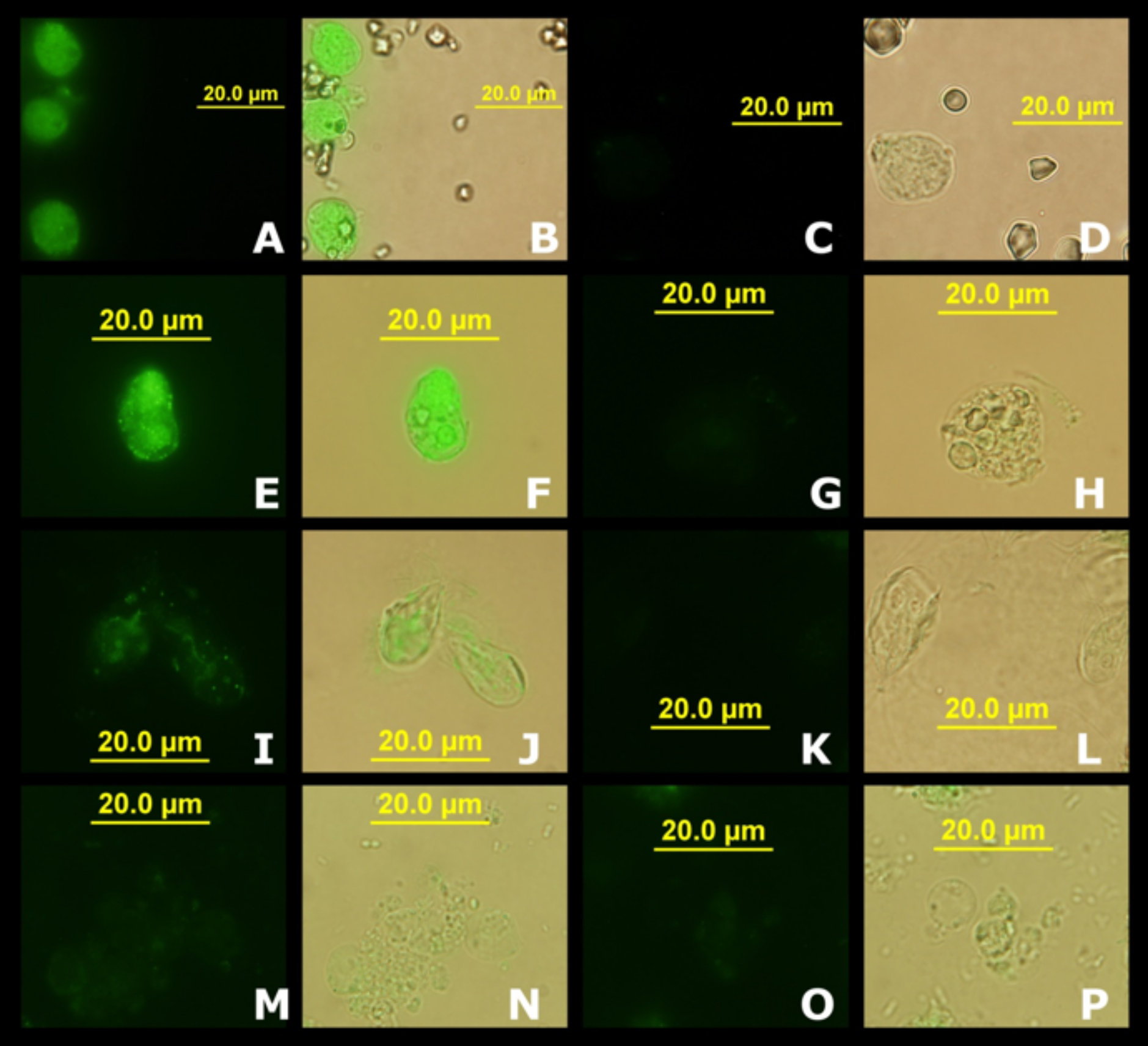


### ​​**Figure S4. Indirect fluorescent antibody (IFA) staining of other species of intestinal protozoa.**

(A) Fluorescent micrograph of *E. moshkovskii* stained with antibody A. (B) Overlay of panel A on the corresponding bright field micrograph. (C) Fluorescent micrograph of *E. moshkovskii* stained with antibody B. (D) Overlay of panel C on the corresponding bright field micrograph. (E) Fluorescent micrograph of *E. histolytica* stained with antibody A. (F) Overlay of panel E on the corresponding bright field micrograph. (G) Fluorescent micrograph of *E. histolytica* stained with antibody B. (H) Overlay of panel G on the corresponding bright field micrograph. (I) Fluorescent micrograph of *G. lamblia* stained with antibody A. (J) Overlay of panel I on the corresponding bright field micrograph. (K) Fluorescent micrograph of *G. lamblia* stained with antibody B. (L) Overlay of panel K on the corresponding bright field micrograph. (M) Fluorescent micrograph of *Blastocystis* sp*.* stained with antibody A. (N) Overlay of panel M on the corresponding bright field micrograph. (O) Fluorescent micrograph of *Blastocystis* sp*.* stained with antibody B. (P) Overlay of panel O on the corresponding bright field micrograph. All slides were prepared alongside negative control slides treated with only the FITC conjugated secondary antibody. Negative control slides exhibited no fluorescence. Note that IFA assays prepared with antibody B did not exhibit fluorescence for these protozoa, while assays prepared using antibody A exhibit fluorescence for *Entamoeba* (diffuse staining) and *Giardia* (punctate staining of certain cellular components), but not *Blastocystis*.

### Table S2. Historic slides from the Natural History Museum, London

| **Specimen Type** | **Catalog Number** | **Collection** | **Scientific Name** | **Year** |
| --- | --- | --- | --- | --- |
| Preserved Specimen | 1987.4.4.15262 | ZOO | *Dientamoeba fragilis* | 1934 |
| Preserved Specimen | 1987.4.4.15261 | ZOO | *Dientamoeba fragilis* | 1934 |
| Preserved Specimen | 1987.4.4.12353 | ZOO | *Dientamoeba fragilis* | Unknown |
| Preserved Specimen | 1987.4.4.13180 | ZOO | *Dientamoeba fragilis* | Unknown |
| Preserved Specimen | 1987.4.4.11321 | ZOO | *Dientamoeba fragilis* | 1909 |
| Preserved Specimen | 1987.4.4.2047 | ZOO | *Dientamoeba fragilis* | Unknown |
| Preserved Specimen | 1987.4.4.15258 | ZOO | *Dientamoeba fragilis* | 1910 |
| Preserved Specimen | 1987.4.4.12335 | ZOO | *Dientamoeba fragilis* | Unknown |
| Preserved Specimen | 1987.4.4.1916 | ZOO | *Dientamoeba fragilis* | 1934 |
| Preserved Specimen | 1987.4.4.15263 | ZOO | *Dientamoeba fragilis* | 1934 |
| Preserved Specimen | 1987.4.4.4781 | ZOO | *Dientamoeba fragilis* | 1893 |
| Preserved Specimen | 1987.4.4.15259 | ZOO | *Dientamoeba fragilis* | 1924 |
| Preserved Specimen | 1987.4.4.15260 | ZOO | *Dientamoeba fragilis* | Unknown |
| Preserved Specimen | 1987.4.4.2046 | ZOO | *Dientamoeba fragilis* | Unknown |
| Preserved Specimen | 1987.4.4.2019 | ZOO | *Dientamoeba fragilis* | Unknown |
| Preserved Specimen | 1987.4.4.1955 | ZOO | *Dientamoeba fragilis* | 1920 |
| Preserved Specimen | 1987.4.4.2020 | ZOO | *Dientamoeba fragilis* | Unknown |

### Table S3. Dimensions of cysts and pre-cysts observed in CDC Slide F (Figure 2)

| **ID** | **Stage** | **Dimensions (µm)** | **Notes** |
| --- | --- | --- | --- |
| F_C1 | Cyst | 5.51 x 5.65 |  |
| F_C2 | Cyst | 4.33 x 4.40 | Uninucleate form. |
| F_PC1 | Pre-Cyst | 6.17 x 6.55 |  |

**Notes:** Trichrome stained slide labelled “FII-25”, included in a training/demonstration set. Unknown year; at least 20 years old. Slides showed abundant trophozoites (~11.7/oil immersion field), and pre- and true cysts.

### Table S4. Dimensions of cysts and pre-cysts observed in CDC slide series IU (Figure 2)

| **ID** | **Stage** | **Dimensions (µm)** | **Notes** |
| --- | --- | --- | --- |
| IU_C1 | Cyst | 6.17 x 6.39 |  |
| IU_C2 | Cyst | 5.30 x 5.40 |  |
| IU_C3 | Cyst | 5.53 x 5.90 |  |
| IU_PC1 | Pre-Cyst | 7.04 x 7.27 | Image also displays a *D. fragilis* trophozoite and *Blastocystis* |
| IU_PC2 | Pre-Cyst | 5.77 x 6.41 |  |

**Notes:** Two Trichrome stained slides from the Lawrence Ash historical collection labelled “Indiana University – Clinical Microbiology”. Unknown year; likely 1980-1990. Slides showed many trophozoites (~4.3 / oil immersion field), pre-cysts, and a small proportion of possible true cysts; *Blastocystis* was also present.

# Supplementary References

**1** Wang J, Wang K, Chen D, et al. Cloning and Characterization of Surface-Localized alpha-Enolase of Streptococcus iniae, an Effective Protective Antigen in Mice. *Int J Mol Sci* 2015; **16**(7): 14490-510.

**2** Mundodi V, Kucknoor AS, Alderete JF. Immunogenic and plasminogen-binding surface-associated alpha-enolase of Trichomonas vaginalis. *Infect Immun* 2008; **76**(2): 523-31.

**3** Song Z, Li Y, Liu Y, Xin J, Zou X, Sun W. alpha-Enolase, an adhesion-related factor of Mycoplasma bovis. *PLoS One* 2012; **7**(6): e38836.

**4** Jenikova G, Hruz P, Andersson MK, et al. Alpha1-giardin based live heterologous vaccine protects against Giardia lamblia infection in a murine model. *Vaccine* 2011; **29**(51): 9529-37.

**5** Carrero JC, Petrossian P, Acosta E, Sánchez-Zerpa M, Ortiz-Ortiz L, Laclette JP. Cloning and characterization of Entamoeba histolytica antigens recognized by human secretory IgA antibodies. *Parasitology Research* 2000; **86**(4): 330-4.

**6** Barratt JL, Cao M, Stark DJ, Ellis JT. The Transcriptome Sequence of Dientamoeba fragilis Offers New Biological Insights on its Metabolism, Kinome, Degradome and Potential Mechanisms of Pathogenicity. *Protist* 2015; **166**(4): 389-408.

**7** Stothard P. The sequence manipulation suite: JavaScript programs for analyzing and formatting protein and DNA sequences. *Biotechniques* 2000; **28**(6): 1102, 4.

**8** Tusnady GE, Simon I. Principles governing amino acid composition of integral membrane proteins: application to topology prediction. *J Mol Biol* 1998; **283**(2): 489-506.

**9** Kolaskar AS, Tongaonkar PC. A semi-empirical method for prediction of antigenic determinants on protein antigens. *FEBS Lett* 1990; **276**(1-2): 172-4.

**10** Larsen J, Lund O, Nielsen M. *Immunome Research* 2006; **2**(1).

**11** Saha S, Raghava GP. Prediction methods for B-cell epitopes. *Methods Mol Biol* 2007; **409**: 387-94.

**12** Barratt JL, Banik GR, Harkness J, Marriott D, Ellis JT, Stark D. Newly defined conditions for the in vitro cultivation and cryopreservation of Dientamoeba fragilis: new techniques set to fast track molecular studies on this organism. *Parasitology* 2010; **137**(13): 1867-78.

**13** Roberts T, Barratt J, Harkness J, Ellis J, Stark D. Comparison of microscopy, culture, and conventional polymerase chain reaction for detection of blastocystis sp. in clinical stool samples. *Am J Trop Med Hyg* 2011; **84**(2): 308-12.

# Appendix A. Enolase sequences identified in the transcriptome

>CONTIG_582

AAATTAAAGGGAATTTTTACTCACTGAGCATTTCATTTTCCTTAAGATATTCGTAAATTT

GGAGAAGTCTTGTGTACTTTTGGATACGTTCACCACGTGCTAATGAACCTGTCTTAATGT

ATTGAGCGCCAATGGCAACTGATAAATCAGCAATGAGAGAATTGCATGTTTCACCAGAAC

GGTGTGAAACCATAACCTTTTGACCAGCTTCTAAAACTAATCTTGCTGCTCTCATAGCTT

CAGTGATTGTACCGATTTGGTTAACCTTGAGAAGAAGAGCGTTGCACCACTTTTCTTTGA

GACCTCTTTGGATTGTCTTTGGATTTGTTGTATAAAGATCATCACCAACTAATTGAATCT

TATCACCAATTTCCTTGTTCATCTTAATCCATGTCTTGTAATCCTTTTCATCAAGGCCAT

CTTCGATTGAAACAATAGCTGGATGCTTGGCAATTAAATCCTTATAATATTGAATCATTT

CATCACCTGTCTTCTTGACACCAACTTCAATTTCATAAAGCTTTGTTGTTTCATCATAAA

ATTCGGAAGCAGCAGCATCAATACCTAACTTAACATCTTCAACTGGCTTGTAACCGGCTT

CAACAATAGCACGTTCGATGTATGTGAGGGCTTCTTCTGGTGTGTCTAAAAGTGGAGCAT

AACCACCTTCATCACCAAGGTTCTTAGCTGAAACACCCTTTTCCTTGGCTAATATGTTAC

CGAGAACATGGTAAATTTCAGCAACCATTCTTAATTGTTCTGGGAATGGAAGATCTTCAC

GTGGAGCAATCATGAATTCTTGGATCTTAAGCTTGCCACCTGCATGCTTACCACCGTTTA

AGATGTTGCAGAATGGTGTTGGTAACTTGAACTTTTCTGGTACTTCGTTGTGATATTGTC

TGGCAAGGTAAAGGAATAATTCTTCGTTGTTCAAATTAGCAGCTGCTGTGGCTAAGGCAA

ATGATGATGCTGTTGTTGCGTTGCCACCAACTTTTTCCTTCAACTCTGAGTCCTTGTTGC

TGGCTAATCTGCAGATTGCTCTGTCCAAATCTTGAAGTGATTCGAATGACATTTGCTTCA

AGGCTGGTGAGATGATCTTTGTGACGTTCTCGGCGGCTGTGACTGTTCCCTTACCGCAGT

ACTTCTTTGGATCGCCATCTCTGAGTTCCTTTGCTTCGTTGGAACCTGTTGATGCACCGC

TTGGAGCTGACGAGCAACCTGCCATAACAACTTCACCAAGATGCTTGGCATAAACATCGA

CTTCAACTGTTGGGTTGCCACGTGAATCGAGAACTTGGCGTCCAACAAGGTGGTCAACAA

CTGGTGGAAGTGCACGGTTCTTCATGAGAGTTGAAAGAACACCAGCAGCATCTTCTGGCT

TGTTTTCAAGGAGAATGTTGACTGCTTCTTGCAAAACTTCACGGATTTTGTATTGTTCGA

GGTATTCTTGTGTCTTTTGTGCAGGAGTAAGTTCTTGGGTTGACATAACTCAAT

>CONTIG_9

AATTGGTGAAACAACAGATCCAGTTGGTACACTCTCACGCCTTCTCAAGGCTAAGGCCCC

AGCTCCAACAATTGATCATCTTGTTGGTCGTGAAGTTCTTGATTCACGTGGTAACCCAAC

AGTTGAAACAGATGTTTATGTTAACTACCTCGGTACAGTTATGTTTGCTGGTCGCTCATC

AGCTCCATCAGGTGCCTCAACAGGTTCAAACGAAGCTCGTGAATTACGTGATGGTGGCAA

GAGATACGGTGGTAAGGGTACACAAAAGGCCGCTGCCAACGTTACAAACATCCTTTCCCC

AGCTCTCAAGGGTATGAAGCTCGATGACCTTAAGGCTATCGATGCCAAGATCTGCGCTAC

AGATGGTACAGAACTTAAGGAACGTGTTGGTGGTAACGCATGCACAGCTACATCATTCGC

TATTGCTGAAGCCGCTGCTGCTCTTGAAGAAATCCCACTCTTCCTTTACTTCGCTCGTCA

ATACTTCGGTGAACAAAACATGCCAAAGAAGTACAAGCTCCCACAATGCTGCTTCAACAT

CCTCAACGGTGGTAAGCATGCCGGTGGTAACCTTAAGATCCAAGAATTCATGGCTTCACC

AGCTAAGGGTACACCACTCCCAGAACAATTAAGAATGGTTGCTGAAGTTTACCAAAAGCT

CGGTGCCATCCTTGTTAAGGAAAAGGGTGTTTCAGCCAAGAACCTTGGTGATGAAGGTGG

TTTCGCTCCATGCCTTGATACACCAGATGAAGCTATTTCATACATTGAACAAGCTATGAA

GGCTGCTGGTTACGAACCAGGTAAGGATATCGGTCTTTGCCTTGATGCTGCTGCCTCAGA

ATTCTATGATGCCGAAACAAAGCTTTACGAAGTTGAAATTGGTGTTAAGAAGACAGGTGA

TGAAATGATCCAATACTGGGCTGATCTTATTGCTAAGCACCCATGCATCATCTCAATCGA

AGATGGTCTTGATGAAAAGGATTATGAACATTGGGCTAAGCTCAACGCCGCTCTCGGTGA

CAAGGTTCAACTTGTTGGTGATGATCTTTACACAACAAATCCAAAGACAATCGCTAAGGG

TATCGAAGGTAAGTGGTGCAATGCTTTACTTCTTAAGGTTAACCAAATCGGTACAATCAG

CGAAGCCATGCAAGCTGCTAAGCTTGTTCTTGATGCTCACCAAAACGTTATGGTTTCACA

CCGTTCTGGTGAAACATGCAACTCAGTTATTGCTGATCTTGTTGTTGGTATTGGTGCTCA

ACAAATCAAGACTGGTTCAACAGCTCGTGGAGAACGTATCCAAAAGTACACACGTCTCCT

TCAAATTTACGAATATCTTAAGGAACACGATATGCTCGAAGAATAAATAAATAAAACA

>CONTIG_13

TTATTCTTCGAGCATATCGTGTTCCTTAAGATATTCATAGATTTGAAGAAGACGTGTGTA

CTTTTGAATACGTTCTCCACGAGCTGTAGAACCTGTCTTGATTTGTTGAGCACCAATGGC

AACAGCAAGATCAGCAATAACTGAGTTGCATGTTTCACCAGAACGGTGTGAAACCATAAC

ATTTTGATGGGCATCAAGGATGAGCTTAGCAGCTTGCATGGCTTCACTGATTGTACCGAT

TTGGTTAACCTTGAGAAGTAAGGCATTGCACCACTTACCTTCAATACCCTTAGCGATTGT

CTTTGGATTTGTTGTGTAAAGATCATCACCAACAAGTTGAACCTTGTCACCGATTGTTTC

GTTAAGCTTAGCCCAATGTTCATAATCCTTTTCGTCAAGACCATCTTCAATTGAAACAAT

GCATGGGTGCTTGGCAATGAGATCAGCCCAGTATTGGATCATTTCATCACCTGTCTTCTT

AACATCAACTTCAACTTCGTAAAGCTTTGTTTCAGCATCATAGAATTCTGAAGCAGCGGC

ATCAAGACCTATTCTGATATCTTTGCCTGGTTCGTAACCAGCTTCCTTGATAGCTTCTTC

AATGATTGAAATAGCTTCTTCTGGTGTGTTAAGGAGTGGAGCGAAACCACCTTCATCACC

AAGGTTCTTAGCGGATAAACCATACTTCTTAACTAAGAGACCACCAAGCTTTTGATAAAC

TTCGGCAACCATACGTAATTGATCTGGGAATGCAATTCCTGGAGCTGGTGAAGCCATGAA

TTCTTGTAACTTAAGGTTACCACCAGCGTGCTTACCACCATTTAAGATGTTGAAGAATGG

TGTTGGTAACTTGAACTTCTTTGGAACGTTTTCAGCACCATAGTATTGTCTAGCTAAGTA

TTCGAATAATTGAAGACCTTCGAGTTCAGCTCCTGTTTCAGCGAGTGCGAATGATGTAGC

AGTGCAAGCATTACCACCAACGTTTTCCTTTAATTCTGTGCCATCAGCCTTGCAGATTTT

TTCATCTAATTCCTTTAAGTTATCTAACTTCTTACCTTCTAATGCCTTTGAAAGGATTTC

AGCAACATTATGAGCAGCCTTTTGTGTGCCCTTGCCTTGGTAACGTGGTGAGTCAGAATC

ACGAAGTTCACGAGCTTCGTTTGATCCTGTTGAGGCACCAGATGGAGCAGATGAACGACC

GGCAAATTGAACTTGACCAAGATACTTAACGTAAACATCAGTTTCAACTGTTGGGTTACC

ACGTGAGTCGAGAACTTCACGGCCAACAAGGTGATCAATAACTGGTGCAGCAGCCTTGGT

CTTGAAGAAACGTGCTAAGAAACCAAAGGCATCTTCTGGCTTTGTGGCAAGCATTTCGTT

CATTGCTTCTTGAAGAAGTTCTGTGATTTTGTGTTCTTGAAGATATTCAGCGGAAGCAGC

TTTGTTATCAGCCATCAAAAAAAGTGAAA

>CONTIG_4111

CAAAATCTTGACCAAATCATTGCTGAATGCAAGGCTNAAATTGGTGAAACAACAGATCCA

GTTGGTACACTCTCACGCCTTCTCAAGGCTAAGGCCCCAGCTCCAACAATTGATCATCTT

GTTGGTCGTGAAGTTCTTGATTCACGTGGTAACCCAACAGTTGAAACAGATGTTTATGTT

AACTACCTCGGTACAGTTATGTTCGCTGGTCGCTCATCAGCTCCATCAGGTGCCTCAACA

GGTTCAAACGAAGCTCGTGAATTACGTGATGGTGGCAAGAGATACGGTGGTAAGGGTACA

CAAAAGGCTGCTGCCAACGTTACAAACATCCTTTCCCCAGCTCTCAAGGGTATGAAACTC

GATGATCTTAAGGCCATTGATGCCAAGATCTGCGCTACAGATGGTACAGAACTTAAGGAA

CGTGTTGGTGGTAACGCATGCACAGCTACATCATTCGCTATTGCTGAAGCCGCTGCTGCT

CTTGAAGAAATCCCACTCTTCCTTTACTTCGCTCGTCAATACTTCGGTGAACAAAACATG

CCAAAGAAGTACAAGCTCCCACAATGCTGCTTCAACATCCTCAACGGTGGTAAGCACGCC

GGTGGTAACCTTAAGATCCAAGAATTCATGGCTTCACCAGCTAAGGGTACACCACTCCCA

GAACAATTAAGAATGGTTGCTGAAGTTTACCAAAAGCTCGGTGCCATCCTTGTTAAGGAA

AAGGGTGTTTCAGCCAAGAACCTTGGTGATGAAGGTGGTTTCGCTCCATGCCTTGATACA

CCAGATGAAGCTATCTCATACATTGAACAAGCTATGAAGGCTGCTGGTTACGAACCAGGT

AAGGATATCGGTCTTTGCCTTGATGCTGCTGCCTCAGAATTCTATGATGCCGAAACAAAG

CTTTACGAAGTTGAAATTGGTGTTAAGAAGACAGGTGATGAAATGATCCAATATTGGGCT

GATCTTATCGCTAAGCACCCATGCATCATCTCAATCGAAGATGGTCTTGATGAAAAGGAT

TATGAACATTGGGCTAAGCTCAACGCCGCTCTCGGTGACAAGGTTCAACTTGTTGGTGAT

GATCTTTACACAACAAATCCAAAGACAATCGCTAAGGGTATCGAAGGTAAGTGGTGCAAT

GCTTTACTTCTTAAGGTTAACCAAATCGGTACAATCAGCGAAGCCATGCAAGCTGCTAAG

CTTGTTCTTGATGCTCACCAAAACGTTATGGTTTCACACCGTTCTGGTGAAACATGCAAC

TCAGTTATTGCTGATCTTGTTGTTGGTATTGGTGCTCAACAAATCAAGACTGGTTCAACA

GCTCGTGGAGAACGTATCCAAAAGTACACACGTCTCCTTCAAATTTACGAATATCTTAAG

GAACACGACATGCTCGAAGAATAAATAAAATAAAATTATTAAGC

# Appendix B. Translated protein sequences

>CONTIG_9/CONTIG_4111 (ENO1)

IGETTDPVGTLSRLLKAKAPAPTIDHLVGREVLDSRGNPTVETDVYVNYLGTVMFAGRSS

APSGASTGSNEARELRDGGKRYGGKGTQKAAANVTNILSPALKGMKLDDLKAIDAKICAT

DGTELKERVGGNACTATSFAIAEAAAALEEIPLFLYFARQYFGEQNMPKKYKLPQCCFNI

LNGGKHAGGNLKIQEFMASPAKGTPLPEQLRMVAEVYQKLGAILVKEKGVSAKNLGDEGG

FAPCLDTPDEAISYIEQAMKAAGYEPGKDIGLCLDAAASEFYDAETKLYEVEIGVKKTGD

EMIQYWADLIAKHPCIISIEDGLDEKDYEHWAKLNAALGDKVQLVGDDLYTTNPKTIAKG

IEGKWCNALLLKVNQIGTISEAMQAAKLVLDAHQNVMVSHRSGETCNSVIADLVVGIGAQ

QIKTGSTARGERIQKYTRLLQIYEYLKEHDMLEE

>CONTIG_13 (ENO2)

MADNKAASAEYLQEHKITELLQEAMNEMLATKPEDAFGFLARFFKTKAAAPVIDHL

VGREVLDSRGNPTVETDVYVKYLGQVQFAGRSSAPSGASTGSNEARELRDSDSPRYQGKG

TQKAAHNVAEILSKALEGKKLDNLKELDEKICKADGTELKENVGGNACTATSFALAETGA

ELEGLQLFEYLARQYYGAENVPKKFKLPTPFFNILNGGKHAGGNLKLQEFMASPAPGIAF

PDQLRMVAEVYQKLGGLLVKKYGLSAKNLGDEGGFAPLLNTPEEAISIIEEAIKEAGYEP

GKDIRIGLDAAASEFYDAETKLYEVEVDVKKTGDEMIQYWADLIAKHPCIVSIEDGLDEK

DYEHWAKLNETIGDKVQLVGDDLYTTNPKTIAKGIEGKWCNALLLKVNQIGTISEAMQAA

KLILDAHQNVMVSHRSGETCNSVIADLAVAIGAQQIKTGSTARGERIQKYTRLLQIYEYL

KEHDMLEE

>CONTIG_582 (ENO3)

MSTQELTPAQKTQEYLEQYKIREVLQEAVNILLENKPEDAAGVLSTLMKNRALPPVVD

HLVGRQVLDSRGNPTVEVDVYAKHLGEVVMAGCSSAPSGASTGSNEAKELRDGDPKKYCG

KGTVTAAENVTKIISPALKQMSFESLQDLDRAICRLASNKDSELKEKVGGNATTASSFAL

ATAAANLNNEELFLYLARQYHNEVPEKFKLPTPFCNILNGGKHAGGKLKIQEFMIAPRED

LPFPEQLRMVAEIYHVLGNILAKEKGVSAKNLGDEGGYAPLLDTPEEALTYIERAIVEAG

YKPVEDVKLGIDAAASEFYDETTKLYEIEVGVKKTGDEMIQYYKDLIAKHPAIVSIEDGL

DEKDYKTWIKMNKEIGDKIQLVGDDLYTTNPKTIQRGLKEKWCNALLLKVNQIGTITEAM

RAARLVLEAGQKVMVSHRSGETCNSLIADLSVAIGAQYIKTGSLARGERIQKYTRLLQIY

EYLKENEMLSE
